# Stimulation of bone formation in cortical bone of the mice treated with a novel bone anabolic peptide with osteoclastogenesis inhibitory activity

**DOI:** 10.1186/ar3621

**Published:** 2012-02-09

**Authors:** Yuriko Furuya, Kohji Uchida, Hisataka Yasuda

**Affiliations:** 1 Nagahama Institute for Biochemical Science, Oriental Yeast Co Ltd, Shiga, Japan; 2 Planning & Development Group Bioindustry Division, Oriental Yeast Co Ltd, Tokyo, Japan

## Background

Receptor activator of nuclear factor-κB ligand (RANKL), a member of tumor necrosis factor (TNF)-α, is produced by osteoblasts (Obs) and stimulates its receptor RANK on osteoclast (Oc) progenitors to differentiate them to osteoclasts. WP9QY peptide designed to mimics TNF receptor's contact site to TNF-α was known to abrogate osteoclastogenesis in vitro by blocking RANKL-RANK signaling. WP9QY ameliorated collagen-induced arthritis and osteoporosis in mouse models. Here we report that the peptide surprisingly exhibited bone anabolic effect in vitro and in vivo.

## Materials and methods

WP9QY was administered subcutaneously to mice three times per day for 5 days at a dose of 10 mg/kg in normal mice, followed by peripheral quantitative computed tomography (pQCT) and histomorphometrical analyses. To clarify the mechanism by which the peptide exerted the bone anabolic effect, we examined the effects of the peptide on osteoblast (Ob) differentiation/mineralization with mouse MC3T3-E1 (E1) cells and human mesenchymal stem (MSC) cells, and those on osteoclast (Oc) differentiation with RAW264 cells in the presence of sRANKL.

## Results

WP9QY augmented bone mineral density (BMD) significantly in cortical bone not in trabecular bone. Histomorphometrical analysis showed that the peptide had little effect on osteoclasts in distal femoral metaphysis, but markedly increased bone formation rate in femoral diaphysis. The peptide markedly increased alkaline phosphatase (ALP, a marker for Ob) activity in E1 and MSC cell cultures and decreased tartrate-resistant acid phosphatase (TRAP, a marker for Oc) activity in RAW264 cell culture in a dose-dependent manner, respectively. In addition, the peptide stimulated mineralization evaluated by alizarin red staining in E1 and MSC cell cultures. The anabolic effect of WP9QY peptide was enhanced markedly by addition of BMP2.

Increases in mRNA expression of IGF1, collagen type I, and osteocalcin were observed in E1 cells treated with the peptide for 12 and 96 h in GeneChip analysis. Addition of p38 MAP kinase inhibitor reduced ALP activity in E1 cells treated with the peptide, suggesting a signal through p38 was involved in the mechanisms.

## Conclusions

Taken together, the peptide abrogated osteoclastogenesis by blocking RANKL-RANK signaling and stimulated Ob differentiation/mineralization with unknown mechanism in vitro. However, in our experimental conditions the peptide exhibited bone anabolic effect dominantly in vivo. Since the peptide is known to bind RANKL, we hypothesize that the peptide shows the bone anabolic activity with reverse signaling through RANKL on Obs.

